# Factors influencing future liver remnant hypertrophy after portal vein embolization: a mini review

**DOI:** 10.3389/fsurg.2026.1979361

**Published:** 2026-10-14

**Authors:** Yuxuan Lu, Hangcheng Liu, Boyang Wu, Canhong Xiang, Jianping Zeng, Quanyu Guo, Hongwei Lu, Shizhong Yang, Jitao Wang, Jiahong Dong

**Affiliations:** 1 Hepato-Pancreato-Biliary Center, Beijing Tsinghua Changgung Hospital, Key Laboratory of Digital Intelligence Hepatology (Ministry of Education), School of Clinical Medicine, Tsinghua Medicine, Tsinghua University, Beijing, China; 2 Research Unit of Precision Hepatobiliary Surgery Paradigm, Chinese Academy of Medical Sciences, Beijing, China; 3 Department of General Surgery, The Second Affiliated Hospital of Xi'an Jiaotong University, Xi'an, China

**Keywords:** future liver remnant, liver hypertrophy, liver regeneration, liver venous deprivation, major hepatectomy, perihilar cholangiocarcinoma, portal vein embolization, posthepatectomy liver failure

## Abstract

**Background:**

Portal vein embolization (PVE) promotes future liver remnant (FLR) hypertrophy before major hepatectomy, but the response varies substantially among patients. This review summarizes determinants of post-PVE FLR hypertrophy to support preoperative risk stratification and individualized planning.

**Methods:**

Targeted searches of PubMed/MEDLINE, Web of Science, Scopus, Embase, and China National Knowledge Infrastructure (CNKI) through 26 August 2026 combined terms for PVE, liver regeneration or FLR hypertrophy, and predictors or associated factors. Reference lists and the authors’ existing reference collection were also screened, and evidence was narratively synthesized across five domains.

**Results:**

FLR hypertrophy reflects multiple interacting determinants. Advanced age, selected chemotherapy exposures, and liver fibrosis have been associated with weaker regenerative responses. Elevated inflammatory markers and biliary infection may impair hypertrophy, whereas adequate embolization extent and appropriate embolic material selection may improve the response.

**Conclusion:**

FLR hypertrophy after PVE is determined by interacting demographic and clinical, laboratory, hepatic and biliary, tumor-related, and procedure-related factors. Recognizing these determinants may improve patient selection, help predict insufficient hypertrophy, and guide individualized PVE strategies to increase the safety of major hepatectomy.

## 1 Introduction

Major hepatectomy is central to potentially curative treatment for selected primary and secondary liver malignancies, including hepatocellular carcinoma, colorectal liver metastases, and perihilar cholangiocarcinoma (pCCA) (1–5). When the future liver remnant (FLR) is too small or functionally impaired, the risk of posthepatectomy liver failure (PHLF) increases substantially. Reported PHLF incidence ranges from 9.2% to 37.8%, and mortality among patients with severe PHLF may reach 52%–68% (6, 7). Portal vein embolization (PVE) is an established preoperative strategy that promotes FLR hypertrophy and can expand the safety margin for major resection.

PVE selectively occludes portal branches supplying the liver to be resected, redirecting portal flow to the contralateral liver and stimulating compensatory hypertrophy. Since the technique was reported by Makuuchi et al. (8), clinical series and meta-analyses have supported its ability to increase FLR volume and reduce PHLF in appropriately selected patients (9–11). Ribero et al. also demonstrated improvements in hypertrophy and resectability after PVE (12). More recently, combined portal and hepatic vein embolization, commonly termed liver venous deprivation (LVD), has broadened the available strategies for accelerating FLR growth (13).

The hypertrophic response to PVE varies substantially. A systematic review reported insufficient hypertrophy in approximately 10%–30% of patients (14). An inadequate response can preclude resection, increase the risk of PHLF, and prolong the interval during which tumor progression may occur (15). Studies have therefore examined patient, biliary, hepatic parenchymal, tumor, and procedural determinants of FLR growth (16, 17). Differences in study design, factor definitions, timing of volumetry, and statistical methods, however, limit direct comparisons.

The specific aim of this Mini Review is to synthesize and critically appraise current evidence on the demographic and clinical, laboratory, hepatic and biliary, tumor-related, and procedure-related factors that influence FLR hypertrophy after PVE, in order to support patient selection, prediction of insufficient hypertrophy, and optimization of PVE strategies.

## 2 Methods

### 2.1 Review design and search strategy

This Mini Review was prepared as a structured narrative review. To improve transparency, targeted searches were performed in PubMed/MEDLINE, Web of Science, Scopus, Embase, and China National Knowledge Infrastructure (CNKI) through 26 August 2026. The searches combined terms for portal vein embolization, liver regeneration or future liver remnant hypertrophy, and risk factors, predictors, determinants, associations, or correlations, with syntax adapted to each database. Reference lists of relevant reviews and primary studies and the authors' existing reference collection were also screened.

### 2.2 Evidence selection and prioritization

Clinical studies, systematic reviews, guidelines, and selected mechanistic studies relevant to patient-, laboratory-, hepatic and biliary-, tumor-, or procedure-related determinants were prioritized. Because the available evidence on determinants of FLR hypertrophy after PVE consists predominantly of single-center retrospective cohorts and small case series, with few meta-analyses or multicenter studies available, all clinically relevant studies were considered. Given the limited number and heterogeneity of the available studies, findings were synthesized qualitatively, and greater interpretive weight was given to larger and more recent studies.

### 2.3 Evidence appraisal and synthesis

Because this was a narrative Mini Review, study selection was purposive; no registered protocol, duplicate screening, formal risk-of-bias assessment, or meta-analysis was undertaken. Evidence was appraised according to study design, consistency across studies, directness to the clinical question, and applicability to current practice, and quantitative outcome ranges are presented to support interpretation rather than as pooled estimates. During manuscript preparation, GPT-5.6 (OpenAI, San Francisco, CA, USA) was used for English-language editing, consistency checks, and formatting. All scientific content, factual statements, citations, and references were reviewed and verified by the authors, who take full responsibility for the final manuscript.

## 3 Demographic and clinical characteristics

### 3.1 Age

Liver regenerative capacity generally declines with age (18, 19). Kasai et al. identified age as an independent negative predictor of the FLR hypertrophy ratio (*β* = −0.78, *P* = 0.015) (20), and previous reviews have suggested that older patients may exhibit slower hypertrophy (18, 21). In contrast, two small cohorts did not detect a significant age difference between higher- and lower-regeneration groups (22, 23). Age is therefore likely to interact with comorbidity, liver quality, and treatment exposure rather than act as a uniform threshold; larger cohorts are needed to define its independent contribution (24).

### 3.2 Alcohol consumption

Chronic alcohol exposure can cause steatosis, inflammation, and fibrosis that impair liver regeneration, even before overt decompensation develops (25). In one single-center pCCA cohort, a history of alcohol consumption was associated with poor FLR regeneration [odds ratio (OR) = 2.616] (22). The available evidence is limited, however, by retrospective exposure assessment and the absence of multicenter validation. Dose, duration, abstinence, and coexisting metabolic liver disease should be characterized in future studies.

### 3.3 Previous chemotherapy

Previous chemotherapy may influence FLR hypertrophy through regimen-specific hepatotoxicity (26). Kasai et al. reported prior chemotherapy as a negative predictor of the FLR hypertrophy ratio (*β* = −21.2, *P* = 0.007) (20). Potential mechanisms include sinusoidal injury after oxaliplatin-containing therapy and steatohepatitis after irinotecan-containing therapy. These effects are likely to depend on cumulative dose, interval from treatment, and baseline liver quality.

The effect of chemotherapy cannot be considered independently of timing and oncologic benefit. Adjuvant capecitabine improves outcomes after resection of biliary tract cancer (27), whereas a retrospective analysis of 186 patients found that preoperative gemcitabine-cisplatin did not significantly reduce PVE-induced FLR hypertrophy or increase postoperative complications, although the resection rate was lower in the chemotherapy group (61.4% vs. 88.4%) (28). Regimen-specific, prospective data are needed to guide treatment sequencing.

## 4 Laboratory factors

### 4.1 Tumor necrosis factor alpha

Tumor necrosis factor alpha (TNF-α) has a context-dependent role in liver regeneration (29). In a cohort of 62 patients with pCCA, serum TNF-α was higher in the low-regeneration group and remained independently associated with poor FLR growth (OR = 2.781; 95% confidence interval, 1.21–6.35) (22). Transient TNF-α signaling contributes to the initiation of hepatocyte proliferation through nuclear factor kappa B pathways (30, 31), whereas sustained inflammation may promote stellate-cell activation, hepatocyte injury, and apoptosis (32). The proposed biomarker role of TNF-α requires prospective validation and standardized sampling.

### 4.2 Bilirubin

Bilirubin reflects the severity of cholestasis and biliary obstruction. In 59 patients, Kasai et al. found that the highest bilirubin level was an independent negative predictor of the FLR hypertrophy ratio (*β* = −1.48, *P* < 0.001) (20). Two additional cohorts reported higher preoperative total bilirubin in patients with poorer regeneration (22, 23). These observational associations may partly represent the effects of biliary infection, duration of obstruction, and underlying parenchymal injury.

Chang et al. found that adequately drained cholestasis alone did not significantly reduce PVE-induced hypertrophy, whereas bilateral drainage in the presence of inflammation was associated with a weaker response (33). This finding suggests that infection control and effective drainage may be more informative than a single bilirubin threshold. The optimal bilirubin target and timing of PVE remain uncertain.

### 4.3 Alkaline phosphatase

Alkaline phosphatase (ALP) is a marker of cholestasis and biliary injury. Huang et al. found that higher ALP was independently associated with a lower daily FLR hypertrophy rate (t = −2.99, *P* = 0.004) (34), while Liu et al. reported an association with poor regeneration (OR = 2.687) (22). ALP may therefore integrate the severity and duration of cholestasis and peribiliary fibrosis, but it has not been validated as a stand-alone predictor (35).

## 5 Hepatic and biliary factors

### 5.1 Liver fibrosis

Fibrosis is a biologically plausible determinant of impaired regeneration (36). In pCCA, prolonged biliary obstruction and recurrent cholangitis may contribute to secondary biliary fibrosis. Small retrospective cohorts have consistently reported more fibrosis among patients with poor FLR growth, with adjusted ORs of 3.534 and 3.82 in two studies (22, 23). A separate pathological comparison similarly found less fibrosis in patients with normal regeneration (37).

Fibrosis can impair regeneration through excessive extracellular-matrix deposition, sinusoidal capillarization, disturbed portal microcirculation, and sustained transforming growth factor beta signaling (30, 38, 39). A large multicenter study also identified hepatic parenchymal quality as an important component of PVE risk assessment in pCCA (11). Standardized histological or noninvasive fibrosis measurements are needed to quantify this effect.

### 5.2 Hepatic functional reserve

Indocyanine green clearance integrates hepatic perfusion and hepatocyte uptake. Kasai et al. included the indocyanine green clearance rate in a multivariable model of FLR hypertrophy (20). Its independent association was not statistically significant (*P* = 0.112), and the incremental value of adding this measure to volumetry and routine laboratory tests remains uncertain.

### 5.3 Biliary infection

Preoperative biliary infection is common in pCCA and may impair the response to PVE. In one cohort, infection was more frequent in the low-regeneration group and remained independently associated with poor FLR growth (OR = 2.461) (22). Huang et al. likewise reported a lower daily hypertrophy rate in patients with infection (t = −2.07, *P* = 0.04) (34). These findings support infection control before PVE, although residual confounding by drainage complexity and disease extent is likely.

Potential mechanisms include endotoxin-mediated Kupffer-cell activation, sustained inflammatory signaling, suppression of interleukin-6/signal transducer and activator of transcription 3 pathways, and periportal microcirculatory disturbance (30, 40). These mechanisms remain incompletely characterized in patients undergoing PVE.

## 6 Tumor-related factors

Tumor anatomy, burden, and biological behavior may influence FLR hypertrophy directly or through their effects on biliary obstruction, inflammation, portal hemodynamics, and the extent of planned resection. Evidence for these relationships is less mature than evidence for hepatic parenchymal and procedural factors.

The Bismuth-Corlette classification describes the longitudinal extent of pCCA. Type III and IV tumors often require extended hepatectomy because of more extensive biliary involvement (41). In a cohort restricted to type III and IV pCCA, Bismuth-Corlette classification was associated with FLR hypertrophy (*P* = 0.027) (23). This association may reflect differences in obstruction, infection, drainage, and resection volume rather than an independent effect of classification alone.

Across liver malignancies, greater intrahepatic tumor burden has been inversely associated with FLR hypertrophy after PVE (42). PVE can also stimulate growth of residual tumors while inducing contralateral liver regeneration (43). Tumor burden and growth kinetics should therefore be considered when selecting patients and scheduling resection after embolization.

Regional lymph-node metastasis has also been associated with poor FLR regeneration in small pCCA cohorts. Reported ORs were 2.561 and 2.744 in two analyses (22, 23), and a third cohort found a higher nodal-metastasis rate in the low-regeneration group (37).

These associations should not be interpreted as evidence that lymph-node metastasis directly suppresses liver growth. Nodal disease may instead be a marker of advanced tumor burden, systemic inflammation, biliary obstruction, cachexia, or complex hilar anatomy (40, 44–46). Multivariable studies are needed to separate tumor biology from these correlated clinical factors.

## 7 Procedure-related factors

### 7.1 Embolic materials

Common PVE materials include polyvinyl alcohol (PVA) particles, coils, gelatin sponge, n-butyl cyanoacrylate (NBCA), and absolute ethanol (12, 16). Gelatin sponge may permit recanalization when used alone, while ethanol can cause pain and carries a risk of non-target embolization. In 63 patients with pCCA, NBCA was associated with greater FLR hypertrophy than PVA plus coils (47.96% vs. 25.23%, *P* < 0.001), a higher daily growth rate (10.91 vs. 6.79 mL/day, *P* = 0.019), and a greater degree of hypertrophy (12.19% vs. 9.18%, *P* = 0.023) (47).

NBCA is a liquid embolic agent that can penetrate distal portal branches and produce durable occlusion, thereby increasing portal-flow redistribution to the FLR (47). An experimental comparison of four embolic materials also found more complete occlusion with NBCA (48). Clinical comparisons remain heterogeneous, and material selection must also consider operator experience, anatomy, safety, and cost.

A retrospective study comparing iodized oil plus coils with PVA plus coils found no significant difference in FLR hypertrophy, resection, or safety outcomes (49). These results suggest that complete and durable portal occlusion may be more important than any single material in all settings.

### 7.2 Extent of embolization

The extent of embolization determines the magnitude of portal-flow redistribution. Yi et al. reported a moderate positive correlation between the embolized vascular-bed proportion and hypertrophy of the nonembolized liver (r = 0.593, *P* < 0.05) (50). This relationship supports complete embolization of the planned resection territory while avoiding non-target injury.

Technical extensions may further increase the response. Embolization of segment IV branches during right PVE has been associated with greater FLR hypertrophy (51). Sequential ipsilateral hepatic-vein embolization after PVE has also been used to augment growth (52), providing the foundation for contemporary LVD strategies.

### 7.3 Portal hemodynamics

PVE increases portal pressure and redirects flow toward the FLR. In one hemodynamic study, main portal pressure increased from 22.4 to 25.9 cm H₂O, and portal-flow velocity on the nonembolized side peaked at day 7 at 26 ± 9 cm/s (50). The relationship between these early hemodynamic changes and later functional hypertrophy requires prospective study.

Severe portal-vein stenosis can limit inflow to the FLR. In a four-patient pCCA series, portal-vein stenting combined with PVE produced a mean 34% increase in FLR volume and enabled R0 resection in all patients (53). The small sample precludes generalization, but the approach may be useful in selected patients. More broadly, preserving arterial inflow while selectively occluding portal flow may reduce PHLF risk (54).

### 7.4 Liver venous deprivation

LVD combines portal and hepatic vein embolization to produce faster and larger FLR hypertrophy than PVE alone (13, 55). In the multicenter DRAGON 0 cohort, combined PVE and hepatic-vein embolization was associated with a higher resection rate than PVE alone (92% vs. 68%, *P* = 0.007) and longer overall survival (median not reached at 36 months vs. 20 months, *P* = 0.004) (56). Because the survival comparison was observational, randomized or carefully matched studies are needed.

## 8 Discussion and future directions

Figure 1 summarizes the principal factors influencing FLR hypertrophy after PVE and their proposed interrelationships. FLR hypertrophy after PVE is governed by interacting demographic and clinical, laboratory, hepatic and biliary, tumor-related, and procedure-related factors. Fibrosis, biliary infection, embolization completeness, and embolic strategy have the most consistent clinical associations, although much of the evidence remains observational. Findings for age and chemotherapy are inconsistent, while evidence for alcohol exposure, indocyanine green clearance, inflammatory biomarkers, and nodal disease is limited. Preoperative assessment should therefore combine volumetry with liver quality, biliary and infectious status, treatment history, tumor burden, and technical planning rather than rely on a single predictor.

**Figure 1:**
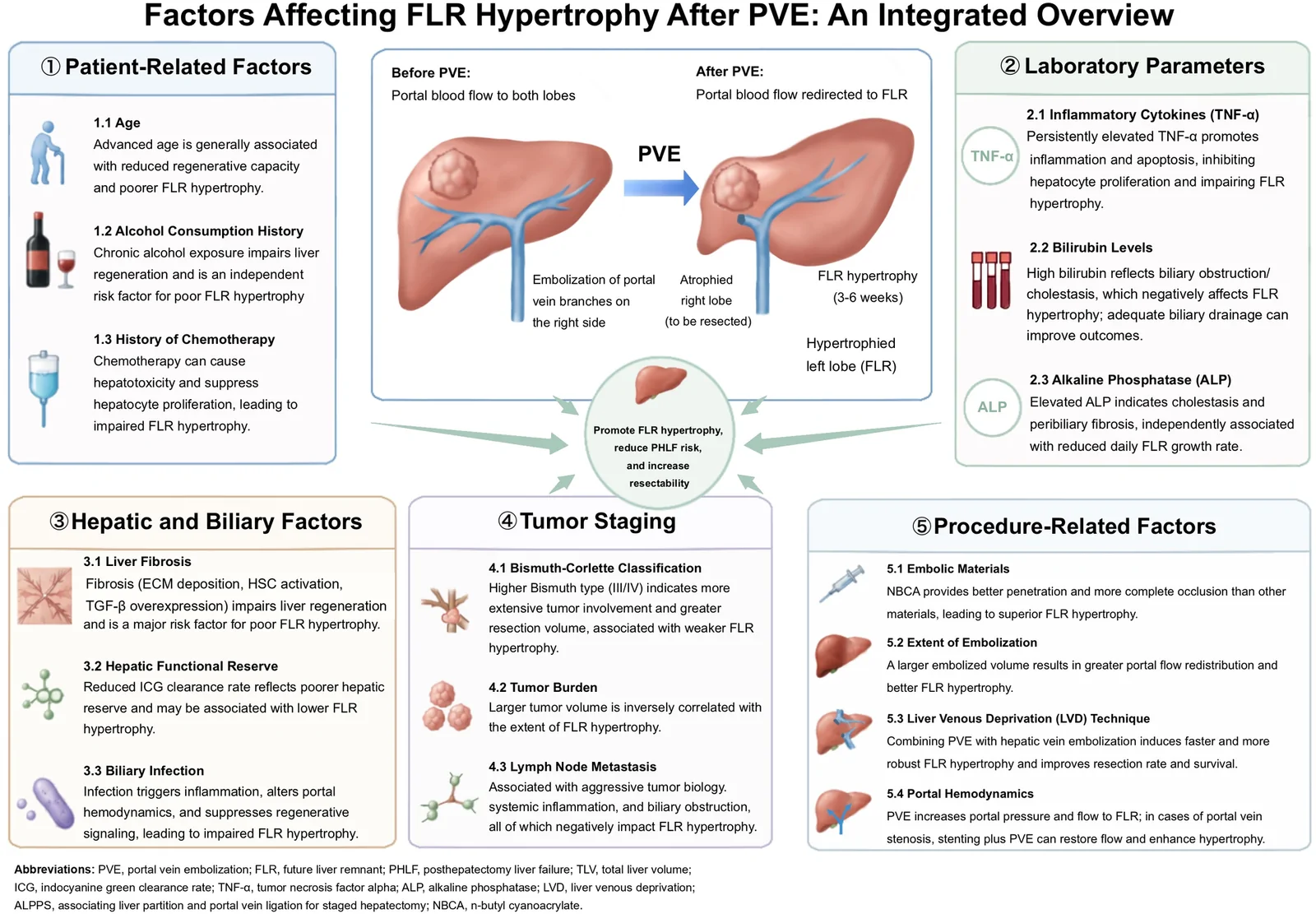
Overview of factors associated with future liver remnant hypertrophy after portal vein embolization and their proposed interrelationships. ALP, alkaline phosphatase; ALPPS, associating liver partition and portal vein ligation for staged hepatectomy; FLR, future liver remnant; ICG, indocyanine green clearance; LVD, liver venous deprivation; NBCA, n-butyl cyanoacrylate; PHLF, posthepatectomy liver failure; PVE, portal vein embolization; TLV, total liver volume; TNF-α, tumor necrosis factor alpha.

The key factors influencing FLR hypertrophy after PVE, the direction of their associations, and representative supporting evidence are summarized in Table 1.

**Table 1:** Summary of factors associated with future liver remnant hypertrophy after portal vein embolization.

Factor domain	Factor	Association with FLR hypertrophy	Representative evidence
Demographic and clinical	Advanced age	Negative predictor of the hypertrophy ratio	Kasai et al. (20); Russolillo et al. (24)
Demographic and clinical	Chronic alcohol exposure	Impaired regeneration (limited clinical evidence)	Seitz et al. (25)
Demographic and clinical	Previous chemotherapy	Regimen-dependent; prolonged chemotherapy may impair hypertrophy	Sturesson et al. (26); Hyakutake et al. (28); Kasai et al. (20)
Laboratory	Elevated TNF-alpha/inflammatory markers	Associated with low regeneration	Liu et al. (22)
Laboratory	Elevated bilirubin	Independent negative predictor	Kasai et al. (20)
Laboratory	Elevated alkaline phosphatase	Lower daily FLR growth rate	Huang et al. (34)
Hepatic and biliary	Liver fibrosis	Impaired regenerative response	Liu et al. (22, 23); Cai et al. (37)
Hepatic and biliary	Biliary infection/cholangitis	More frequent in low-regeneration groups	Liu et al. (22); Chang et al. (33)
Tumor-related	Greater intrahepatic tumor burden	Inverse association with hypertrophy	Takahashi et al. (42)
Tumor-related	Regional lymph-node metastasis	Associated with poor regeneration in small pCCA cohorts	Liu et al. (22, 23); Cai et al. (37)
Procedure-related	Embolic material (e.g., NBCA)	Durable distal occlusion may enhance the response	Huang et al. (47); de Baere et al. (48)
Procedure-related	Greater embolization extent (including segment IV)	Greater FLR hypertrophy	Yi et al. (50); Madoff et al. (51)
Procedure-related	Liver venous deprivation	Faster and larger hypertrophy than PVE alone	Guiu et al. (13); Korenblik et al. (55, 56)

This review has limitations. Although multiple databases were searched, it used a targeted narrative strategy rather than a registered or exhaustive systematic-search protocol and did not include duplicate screening or formal risk-of-bias assessment. Many cited studies are small, retrospective, and focused on pCCA, and definitions and timing of FLR assessment vary. Causal interpretations should therefore be made cautiously.

Future research priorities include:

1. Establish multicenter prospective cohorts using standardized FLR volume, function, and kinetic-growth outcomes at prespecified time points.
2. Test modifiable factors, including infection control, biliary-drainage strategy, bilirubin targets, embolic material, and embolization extent.
3. Develop and externally validate multivariable prediction models that integrate clinical, biochemical, imaging, and functional measures, building on existing PHLF models (57, 58).
4. Compare PVE with accelerated strategies such as LVD and ALPPS using patient-centered outcomes, including resection, PHLF, oncologic progression, and survival (7, 11, 55, 56).

## 9 Conclusions

FLR hypertrophy after PVE is influenced by interacting demographic and clinical characteristics, laboratory factors, hepatic and biliary status, tumor-related features, and procedure-related choices. Among these, liver fibrosis, biliary infection, embolization extent, and embolic strategy show the most consistent associations with the regenerative response, whereas evidence for age, alcohol exposure, chemotherapy, inflammatory biomarkers, and tumor-related factors remains limited or inconsistent.

These findings have direct clinical implications. Preoperative evaluation should combine FLR volumetry with assessment of liver quality, cholestasis and biliary infection, treatment history, tumor burden, and portal hemodynamics rather than relying on any single predictor. For patients at risk of insufficient hypertrophy, modifiable factors should be optimized before PVE, and accelerated strategies such as liver venous deprivation may be considered in selected cases. Standardized outcome definitions and prospective multicenter validation are needed to translate these associations into reliable predictive tools.
