# Correction: Factors influencing future liver remnant hypertrophy after portal vein embolization: a mini review

**DOI:** 10.3389/fsurg.2026.2031592

**Published:** 2026-10-28

**Authors:** Yuxuan Lu, Hangcheng Liu, Boyang Wu, Canhong Xiang, Jianping Zeng, Quanyu Guo, Hongwei Lu, Shizhong Yang, Jitao Wang, Jiahong Dong

**Affiliations:** 1 Hepato-Pancreato-Biliary Center, Beijing Tsinghua Changgung Hospital, Key Laboratory of Digital Intelligence Hepatology (Ministry of Education), School of Clinical Medicine, Tsinghua Medicine, Tsinghua University, Beijing, China; 2 Research Unit of Precision Hepatobiliary Surgery Paradigm, Chinese Academy of Medical Sciences, Beijing, China; 3 Department of General Surgery, The Second Affiliated Hospital of Xi'an Jiaotong University, Xi'an, China

**Keywords:** future liver remnant, liver hypertrophy, liver regeneration, liver venous deprivation, major hepatectomy, perihilar cholangiocarcinoma, portal vein embolization, posthepatectomy liver failure

An incorrect Funding statement was provided. The correct funding statement reads:

This work was supported by the Key Program of the National Natural Science Foundation of China, “Research on the Structural, Functional, and Pathological Characteristics of Hepatic Segments Based on Multimodal Imaging Omics” (grant No. 81930119), and the CAMS Innovation Fund for Medical Sciences (CIFMS), Research Unit of Precision Hepatobiliary Surgery Paradigm (grant No. 2019-I2M-5-056).

The original version of this article has been updated.

